# Differences in college students’ occupational dysfunction and mental health considering trait and state anxiety during the COVID-19 pandemic

**DOI:** 10.7717/peerj.13443

**Published:** 2022-05-19

**Authors:** Yasuaki Kusumoto, Rieko Higo, Kanta Ohno

**Affiliations:** 1 Department of Physical Therapy, Fukushima Medical University School of Health Sciences, Fukushima city, Fukushima, Japan; 2 Department of Societal Management, Faculty of Human Society, Sagami Women’s University, Sagamihara, Kanagawa, Japan; 3 Major of Occupational Therapy, Department of Rehabilitation, Tokyo University of Technology, Ohta-ku, Tokyo, Japan

**Keywords:** Occupational dysfunction, Trait anxiety, State anxiety, COVID-19, College student, Classification and assessment of occupational dysfunction, State-trait anxiety inventory, Profile of mood states 2nd edition, European health literacy survey questionnaire, Stress Response Scale-18

## Abstract

**Background:**

Due to the COVID-19 pandemic, university education has shifted from face-to-face classes to online and distance learning. Effects of exposure may manifest in terms of psychological, cognitive, or musculoskeletal impairments that affect an individual’s daily functioning and quality of life. There is a dearth of studies exploring anxiety states, occupational dysfunction, and mental health associated with the new standard of increased telecommunication. Accordingly, the present study aimed to identify the differences in occupational dysfunction, health literacy, positive and negative emotions, and stress response considering the anxiety states of college students during the COVID-19 pandemic. Another purpose is to identify relationships among the parameters such as occupational dysfunction and mental health.

**Methods:**

This cross-sectional study included 358 students (average age: 18.5 years, age range: 18–29 years). Five tools were used: the State-Trait Anxiety Inventory (STAI), Classification and Assessment of Occupational Dysfunction (CAOD), European Health Literacy Survey Questionnaire (HLS-EU-Q47), Profile of Mood States 2nd Edition (POMS-2), and Stress Response Scale-18 (SRS-18). Based on the cutoff value of state and trait anxiety of the STAI, the participants were classified into four groups and compared using one-way analysis of variance and multiple comparison tests. The relationship between all parameters was analyzed using Pearson’s correlation coefficient.

**Results:**

The group with high trait anxiety and high state anxiety had the highest CAOD total score, Total Mood Disturbance score on the POMS-2, SRS-18 score, and scores on many sub-items of the three parameters. The prevalence of occupational dysfunction was 47% for university students, and there was a variation of from 19 to 61% in each group. The correlation coefficients of the state and trait anxiety scores of the STAI, Total Mood Disturbance score, and SRS-18 ranged from .64 to .75. Additionally, the correlation coefficient between the CAOD total score and these parameters ranged from .44 to .48.

**Conclusion:**

The prevalence of occupational dysfunction was highest in the group with high trait anxiety and high state anxiety, and occupational dysfunction, negative emotions, and stress responses were strongest in this group. Our findings point to potential areas for targeted support and interventions.

## Introduction

It has been reported worldwide that the COVID-19 pandemic has led to an increase in the number of students, regardless of faculty, with generalized anxiety disorder symptoms, depression, and anxiety (García-Espinosa et al., 2021). Studies show a decline in student mental health due to the pandemic; 25–27% of Chinese university students showed significant negative psychological changes (Cao et al., 2020; Li et al., 2020), and 34% and 45% of Pakistani university students reported anxiety and depression, respectively (Salman et al., 2020). In Spain, 21% and 34% of university students reported frequencies of moderate anxiety and depression, respectively (Odriozola-González et al., 2020). In Mexico, the prevalence of stress symptoms was reported to be 32% (González-Jaimes et al., 2020). A study on the rate of anxiety and depression among university students before the COVID-19 pandemic reported that in Saudi Arabia, 3.2% of males and 12.3% of females were moderately anxious, and approximately 10% of both males and females were depressed (Anwar et al., 2021). Additionally, in a four-year longitudinal study in China, 6.7–7.7% of both male and female college students were anxious, and 6.9–8.0% were depressed (Gao, Ping & Liu, 2020). All these studies suggest that the pandemic may have worsened the existing mental health conditions within the student population. Such anxiety and depression are related to excessive stress and increased anxiety due to changes in social life. Notably, students are also less likely to seek psychological help despite having higher rates of depression and mental illness (Chandratre, 2020).

In addition, owing to the COVID-19 pandemic, university education has shifted from face-to-face classes to online modes and distance learning. This mode of communication, performed through remote teaching, is called telecommunication (Mheidly, Fares & Fares, 2020). University class formats are also changing to suit the context in each country, such as full web-based classes and hybrids of face-to-face and web-based classes. However, this change may not be conducive to supporting or enhancing student mental health. Researchers worldwide have focused on the relationship between the use of smart devices and stress (Sansone & Sansone, 2013). Exposure to computers and smartphone screens is associated with several stress-related symptoms (Lemola et al., 2015). Increased computer use among teenagers has been associated with increased anxiety levels (Khouja et al., 2019). Furthermore, increased online activity in a cohort of over 3,000 USA students is relevant to moderate-to-severe depression (Madhav, Sherchand & Sherchan, 2017). Particularly, anxiety is more prevalent among younger students, such as first-and second-year university students. Prolonged exposure to telecommunication can also affect physical health. Additionally, looking at a screen for long hours or hunching over can lead to physical harm (Fares, Fares & Fares, 2017). Physical harm can contribute to psychological, cognitive, or musculoskeletal impairments that affect an individual’s quality of life and daily functioning (Hossmann & Hermann, 2003). It is feared that staring at computer and smartphone screens for long periods will increase stress and anxiety. These telecommunications-related mental health stressors, in addition to other stressors associated with behavioral regulation in society in a pandemic, could eventually lead to malaise, burnout, and psychological problems (Mheidly, Fares & Fares, 2020). There are two ways of thinking about anxiety: “state anxiety” and “trait anxiety” (Spielberger et al., 1983). State anxiety refers to the state of anxiety regarding how you are feeling right now. Trait anxiety refers to the state of anxiety regarding how one normally feels. Both these perspectives are considered important for healthcare professionals to intervene to improve mental health and reduce stress. However, previous research has focused on whether anxiety is high or low, and neither the COVID-19 pandemic studies nor the telecommunication studies have taken the anxiety status into account.

Occupational dysfunction refers to the negative aspects of people’s lifestyles and daily functioning and has received much attention in the rehabilitation field (Teraoka & Kyougoku, 2015b). Occupational dysfunction, proposed in the Model of Human Occupation (Kielhofner, 1995), is defined as a negative experience associated with engaging in or not properly performing daily activities (work, self-care, leisure, and rest) (Kielhofner, 1995; Teraoka & Kyougoku, 2015b). It is recognized as a major health problem worldwide (Kielhofner, 1995; Kielhofner et al., 1999). Occupational dysfunction includes occupational marginalization, occupational imbalance, occupational alienation, and occupational deprivation (Teraoka & Kyougoku, 2015b) and may be present without any obvious medical symptoms (Kyougoku, 2010). Because occupation includes not only business, work, and labor but also many other activities, such as education, activities of daily living, play, social participation, and rest (Teraoka & Kyougoku, 2015b), it is influenced by social conditions and personal circumstances. Therefore, the risk of occupational dysfunction is not limited to adult workers but also people in various stages of development, such as adolescence and old age. Occupational marginalization is defined as a lack of opportunities to engage in the decision-making process in desired daily activities (Townsend & Wilcock, 2004). For example, a client with an illness who wants to get a regular job but is unable to do so because of the illness, or a person who is forced to work on a task that no one appreciates when he does it, is also in a state of occupational marginalization. Occupational imbalance is defined as the state of imbalance when engaging in daily activities (Anaby et al., 2010). For example, taking a lot of time for one task, doing only what you do not want to do, or resting all the time is a type of occupational imbalance. Occupational alienation is defined as a situation in which a client is unable to find meaning and be satisfied in an individual’s internal needs related to their daily activities (Bryant, Craik & McKay, 2004). For example, a client in the hospital living a monotonous life according to a schedule, or a student routinely taking classes, feeling bored with doing something and just kind of doing it. Occupational deprivation is defined as a condition in which a client is unable to perform or engage in a task for external reasons, regardless of the client’s intentions for daily activities (Whiteford, 2000). For example, it is the occupational deprivation for a college student to be unable to watch a movie at a movie theater that he or she had planned to due to a delayed train.

In previous Japanese studies, the prevalence of occupational dysfunction was 15% in healthy older adults (Fujii et al., 2021), 36% in office workers (Akiyama & Kyougoku, 2010), and 75% in healthcare workers without an obvious medical disease (Miyake et al., 2014). A previous study suggested a strong relationship between occupational dysfunction classification and stress response and depression in healthcare workers via confirmatory and exploratory factor analysis and path analysis (Teraoka & Kyougoku, 2015a); therefore, those with occupational dysfunction need additional care to decrease anxiety. When considering occupational dysfunction, it seems important to simultaneously consider mental health conditions such as anxiety and stress. The introduction of distance learning during the pandemic may increase the level of occupational dysfunction because of increased screen time and anxiety due to changes in social conditions. Nevertheless, none have taken occupational dysfunction of college students by considering their anxiety status. There may be differences in the prevalence of occupational dysfunction among college students by considering the anxiety states. Clarifying the differences between occupational dysfunction and mental health by considering college students’ anxiety status, and clarifying the relationship between occupational dysfunction, anxiety states and various mental health parameters, such as emotions and stress, will lead to appropriate mental health care being provided to students with occupational dysfunction. In the future, this will be helpful in selecting appropriate subjects when conducting preventive involvement.

Accordingly, the present study adds to the existing literature by identifying the differences in occupational dysfunction, health literacy, positive and negative emotions, and stress response by considering college students’ anxiety states during the COVID-19 pandemic, in addition to identifying the relationships between such parameters as occupational dysfunction and mental health. In conducting this study, we formulated two research hypotheses. The first is that individuals with higher anxiety states have a higher prevalence of occupational dysfunction and tend to have higher negative emotion, and stress response. Second, occupational dysfunction is significantly correlated with mental health parameters such as negative emotion and stress response.

## Materials & Methods

### Participants

All participants were enrolled at a university in Tokyo and took a hybrid of face-to-face and web-based distance learning classes to avoid the spread of COVID-19. The survey period was from April 12 to May 6, 2021. In line with the social conditions at the time of the survey, Tokyo was under a declared state of emergency from April 25 to May 11. Japan was amid a fourth wave pandemic. In the neighboring prefectures of Saitama, Kanagawa, and Chiba, where the participants lived, the Japanese government had applied priority measures to prevent the spread of the disease.

Since April 2020, face-to-face classes and web-based distance learning have continued to be offered in roughly half of all faculties. A description of the research was given at the end of their lectures. Participants were explained, orally and in writing, that participation or non-participation in this study would not affect the grade determination, and their consent was obtained in writing and also orally. A total of 409 participants without any obvious medical diseases were contacted. Those who submitted incomplete questionnaires (*n* = 39) and those who could not provide consent (*n* = 12) were excluded; 358 participants were included in the analysis. Most participating students were male (*n* = 222) and belonged to the undergraduate department of rehabilitation (*n* = 123) (Table 1). The mean age (standard deviation) of the participants was 18.5 years (1.4), with a range of 18–29 years. The university where the study was conducted granted ethical approval to conduct the study within its facilities (authorization number: E21HS-005).

**Table 1:** Descriptive statistics for the participants’ characteristics (*n* = 358).

Undergraduate, *n*		
Department of rehabilitation	123
School of media science	72
School of engineering	59
School of computer science	56
School of bioscience and biotechnology	48
Sex, *n*		
Male	222
Female	133
Others	3

### Design

This was a cross-sectional study involving college students in Tokyo.

### Measures

The following five tools were used in this study: the State-Trait Anxiety Inventory (STAI), Classification and Assessment of Occupational Dysfunction (CAOD), European Health Literacy Survey Questionnaire (HLS-EU-Q47), Profile of Mood States 2nd Edition (POMS-2), and Stress Response Scale-18 (SRS-18). To account for participant fatigue and measurement time, all participants were measured on two separate occasions within one week. For students in the department of rehabilitation, measurements were performed on April 12, 19 and 26. For students in other departments, measurements were performed on April 15, 22, 29, and May 6. CAOD and HLS-EU-Q47 were measured the first time, and STAI, POMS-2, and SRS-18 were measured the second time.

#### STAI

The Japanese version of the STAI, which has sound reliability and validity, was used to evaluate anxiety states (Tadashi et al., 2021). The internal consistencies (Cronbach’s *α* = .83 –.87) of STAI-J were adequate (Tadashi et al., 2021). The STAI contains 40 items and is a self-report scale assessing the separate dimensions of “trait” (20 items) and “state” anxiety (20 items) (Tadashi et al., 2021; Spielberger et al., 1983). Items are rated on a 4-point Likert scale, and the grading standards of the Trait-anxiety inventory are as follows: 1 = almost none, 2 = some, 3 = often, 4 = almost always. The grading standards of the State-anxiety inventory are as follows: 1 = not at all, 2 = some, 3 = moderate, 4 = very obvious. Positive emotion items are reverse scored. The minimum and maximum scores on the two scales are 20 and 80 points, respectively, with higher scores indicating greater anxiety levels. The inventory provides a cutoff value of 45 and 42 points for trait anxiety and state anxiety, respectively (Tadashi et al., 2021). The authors have permission to use this instrument by purchasing it from the marketer.

#### CAOD

The Japanese version of the CAOD has been widely used to assess occupational dysfunction (Teraoka & Kyougoku, 2013; Teraoka & Kyougoku, 2015a; Teraoka & Kyougoku, 2015b; Teraoka & Kyougoku, 2015c). Sound reliability and validity have been confirmed for use among university students (Kielhofner et al., 1999). The internal consistencies (Cronbach’s *α* = .81–.91) of CAOD were adequate (Teraoka & Kyougoku, 2015c). CAOD comprises four factors, totaling 16 items: occupational imbalance (four items), occupational deprivation (three items), occupational alienation (three items), and occupational marginalization (six items). Items are rated on a 7-point response scale ranging from 1 (disagree) to 7 (agree). The minimum and maximum scores are 16 points and 112 points, respectively, with higher scores indicating stronger levels of occupational dysfunction. The most commonly used cutoff value for healthcare workers is 52 points (Teraoka & Kyougoku, 2013). In this study, this cutoff value was used because no cutoff value has been calculated for college students and because it was expected that college students would be less stressed than medical professionals. This instrument has been posted on the original author’s website (https://mutsumiteraoka.blogspot.com/2016/12/caod.html) and can be used freely.

#### HLS-EU-Q47

The Japanese version of the HLS-EU-Q47, which has good reliability and validity, was used to evaluate health literacy (Nakayama et al., 2015). The internal consistencies (Cronbach’s *α* = .92–.97) of HLS-EU-Q47 were adequate (Nakayama et al., 2015). Health literacy indices are constructed as a general health literacy index (GEN-HL) comprising 47 items as well as three sub-indices: health care health literacy index (HC-HL) comprising 16 items, disease prevention health literacy index (DP-HL) comprising 15 items, and health promotion health literacy index (HP-HL) comprising 16 items. Categories are rated on a 4-point Likert scale (1 = very difficult, 2 = fairly difficult, 3 = fairly easy, 4 = very easy). In this study, we included the response “don’t know/not applicable”; this response was coded as a missing value. Index scores were standardized on a metric between 0 and 50 using the formula: (MEAN-1) × (50/3), where MEAN is the mean of all the item responses for each participant. The average score for Japanese was 25.3 for GEN-HL, 25.7 for HC-HL, 22.7 for DP-HL, and 25.5 for HP-HL, which is 7–11.5 points lower than that of European countries (Nakayama et al., 2015). This instrument appears as an additional file in the original paper (Nakayama et al., 2015), and the authors have permission to use this instrument from the copyright holders.

#### POMS-2

The Japanese version of the POMS-2, which shows sound reliability and validity, was used to evaluate positive and negative emotions (Yokoyama & Watanabe, 2015). The internal consistencies (Cronbach’s *α* = .84–.95) of POMS-2 were adequate (Yokoyama & Watanabe, 2015). The POMS-2 consists of 35 items and comprises seven subscales: “Anger–Hostility” (A–H), “Confusion–Bewilderment” (C–B), “Depression–Dejection” (D–D), “Fatigue–Inertia” (F–I), “Tension–Anxiety” (T–A), “Vigor–Activity” (V–A), and “Friendliness” (F). Items are rated on a 5-point Likert scale ranging from 0 (not at all) to 4 (very), according to the feelings of the participant (Yokoyama & Watanabe, 2015; McNair & Heuchert, 2012). Total mood disturbance (TMD) score = (A−H+C−B+D−D+F−I+T−A) –V-A. Lower scores in A–H, C–B, D–D, F–I, T–A, and TMD and higher scores in V–A and F are interpreted as indicative of optimum emotional state. The authors have permission to use this instrument by purchasing it from the marketer.

#### SRS-18

The SRS-18 was used to evaluate stress responses. It was developed in Japan and confirmed to have good reliability and validity (Suzuki, 2006; Suzuki et al., 1997). The internal consistencies (Cronbach’s *α* = .82–.88) of SRS-18 were adequate (Suzuki et al., 1997). It measures the psychological stress encountered over a short time and comprises 18 items related to daily changes in feelings about events that a typical person encounters daily. It includes three subscales: depression and anxiety (six items), such as sadness and worry, displeasure and anger (six items), and lassitude (six items). Each question indicated how they felt about events related to the three subscales. Items are rated on a 4-point response scale ranging from 0 (completely different) to 3 (correct). Higher total scores indicate higher stress levels. All items are not reverse scored. The minimum and maximum total scores are 0 points and 54 points, respectively, and the minimum and maximum scores on each subscale are 0 points and 18 points, respectively. The authors have permission to use this instrument by purchasing it from the marketer.

### Statistical analysis

First, the normality of all variables was confirmed using the Shapiro–Wilk test. Based on the cutoff values of 45 and 42 points for trait and state anxiety of the STAI (Tadashi et al., 2021), the participants were classified into four groups: high trait anxiety and high state anxiety (Group A), high trait anxiety and low state anxiety (Group B), low trait anxiety and high state anxiety (Group C), and low trait anxiety and low state anxiety (Group D). To investigate the differences between each parameter among the four groups, STAI, CAOD, HLS-EU-Q47, POMS-2, and SRS-18 were examined using one-way analysis of variance and multiple comparison tests using the Bonferroni method. The prevalence of occupational dysfunction for all university students and each group was calculated based on a cutoff value of 52 points of the CAOD from a previous study (Teraoka & Kyougoku, 2013). The ratio of people whose CAOD total was above 52 was examined using chi-squared test. The relationship between all parameters was analyzed using Pearson’s correlation coefficient. All analyses were performed using SPSS statistical package for Windows, version 27.0. software (IBM Corp., Tokyo, Japan). Statistical significance was set at *P* < .05.

## Results

The attributes of the participants are listed in Table 1, and scores on all variables for the four groups are shown in Table 2. In Group A, the CAOD total score, TMD score of POMS-2, SRS-18 score, and many scores of sub-items of the three parameters were the strongest. The prevalence of occupational dysfunction was 47% for university students, and there was a variation in each group, with 61% in group A, 31% in group B, 38% in group C, and 19% in group D.

**Table 2:** Results of the measurements.

Measurements	All	Group A (*n* = 206)	Group B (*n* = 13)	Group C (*n* = 68)	Group D (*n* = 69)	Degrees of freedom	*P* value of one-way analysis of variance
Trait anxiety, point	45.2 (10.9)	55.3 (6.9)	41.6 (2.4)^a^	50.6 (4.3)^a,b^	36.2 (6.2)^*^	3	*p* < .001
State anxiety, point	50.2 (9.7)	52.3 (7.5)	46.7 (5.1)^*^	36.7 (4.5)^a,b^	32.4 (5.8)^*^	3	*p* < .001
CAOD total score, point	50.8 (19.8)	56.7 (18.2)	39.1 (17.2)^a^	49.8 (17.9)^a^	36.9 (18.6)^a,c^	3	*p* < .001
Number of people whose CAOD total is above 52 points, n [%]	168 [47%]	125 [61%]	4 [31%]	26 [38%]	13 [19%]	3	*p* < .001
Occupational imbalance	15.7 (6.4)	17 (5.9)	13.3 (6.2)	16 (6.4)	11.9 (6.4)^a,c^	3	*p* < .001
Occupational deprivation	8.7 (4.7)	10 (4.7)	6.5 (3.6)^a^	8.1 (4.4)^a^	6.3 (4)^a^	3	*p* < .001
Occupational alienation	11.3 (4.9)	12.6 (4.6)	8.5 (4.6)^a^	11.4 (4.4)	7.9 (4.5)^a,c^	3	*p* < .001
Occupational marginalization	15.1 (7.6)	17.2 (7.5)	10.7 (5.4)^a^	14.4 (7)^*^	10.8 (6.8)^a,c^	3	*p* < .001
GEN-HL 0-50	20 (7.5)	21.3 (7.1)	20.5 (11)	18.8 (7.3)	17.1 (7.4)^a^	3	*p* < .001
HC-HL 0-50	20.7 (7.7)	21.6 (7.5)	23.2 (10.8)	19.1 (7.4)	18.9 (7.4)	3	.010
DP-HL 0-50	18.3 (8.7)	19.8 (8.1)	18.9 (11.9)	16.8 (8.3)	15 (8.9)^a^	3	*p* < .001
HP-HL 0-50	20.9 (8.9)	22.3 (8.4)	19.4 (12.6)	20.3 (9)	17.7 (8.9)^a^	3	.002
TMD score, point	25 (18.3)	34 (15.5)	15.1 (16.9)^*^	21.5 (12.1)^a^	4 (10.7)^a,c^	3	*p* < .001
A-H	3.6 (3.7)	4.5 (3.9)	3.1 (4.6)	3 (3)^a^	1.7 (2.5)^a^	3	*p* < .001
C-B	6 (3.7)	7.4 (3.5)	3.3 (3.1)^a^	5.7 (2.9)^a^	2.8 (2.2)^a,c^	3	*p* < .001
D-D	6.1 (4.6)	8 (4.5)	3.3.4 (4.3)^a^	5.5 (3.7)^a^	2 (2.1)^a,c^	3	*p* < .001
F-I	8.3 (4.2)	9.8 (3.9)	6.4 (4.1)^a^	8.1 (3.8)^a^	4.5 (2.9)^a,c^	3	*p* < .001
T-A	9.1 (4.6)	10.8 (4.1)	6.5 (4.7)^a^	8.1 (3.9)^a^	5.2 (3.4)^a,c^	3	*p* < .001
V-A	8.2 (4.5)	6.6 (3.9)	7.6 (3.2)	8.8 (3.9)^a^	12.3 (4.4)^*^	3	*p* < .001
F	10.4 (3.9)	9.5 (3.7)	9.5 (2.8)	10.8 (3.4)	12.8 (4)^*^	3	*p* < .001
SRS-18 total score, point	16.5 (11.1)	21.4 (10)	9.9 (12.6)^a^	13.3 (8.5)^a^	6.4 (6.4)^a,c^	3	*p* < .001
Depression-Anxiety	6 (4.4)	8 (4.2)	3.2 (4)^a^	4.6 (3.4)^a^	2.2 (2.6)^a,c^	3	*p* < .001
Displeasure and anger	3.4 (3.6)	4.5 (3.8)	2.8 (4.7)	2.3 (2.8)^a^	1.3 (1.8)^a^	3	*p* < .001
Lassitude	7 (4.5)	8.9 (3.9)	3.9 (4.4)^a^	6.4 (3.9)^a^	2.9 (2.9)^a,c^	3	*p* < .001

**Notes.**

Average (standard deviation); Group A: high trait anxiety and high state anxiety; Group B: high trait anxiety and low state anxiety; Group C: low trait anxiety and high state anxiety; Group D: low trait anxiety and low state anxiety.

a group A *vs*. group B, C, and D.

b group B *vs*. group C and D.

c group C *vs*. group D.

* *p* < .05.

Between groups C and D, which had low trait anxiety, group C had stronger CAOD, POMS-2, and SRS-18 score. The HLS-EU-Q47 score was lower in group D than in group A.

The results of the correlation analyses between the parameters are listed in Table 3. The correlation coefficients among the trait and state anxiety scores of the STAI, TMD score, and SRS-18 ranged from .64 to .75. The correlation coefficient between the total CAOD score and these parameters ranged from .44 to .48. The correlation coefficients between the GEN-HL scores on HLS-EU-Q47 and other parameters ranged from .14 to .24.

**Table 3:** Results of the correlation analysis between the parameter.

	Trait anxiety	State anxiety	CAOD total score	Occupational imbalance	Occupational deprivation	Occupational alienation	Occupational marginalization	GEN-HL 0–50	HC-HL 0–50	DP-HL 0–50	HP-HL 0-50	TMD score	A–H	C–B	D–D	F–I	T–A	V–A	F	Total score	Depression– Anxiety	Displeasure and anger
State anxiety	.76^*^																					
CAOD total score	.48^*^	.44^*^																				
Occupational imbalance	.35^*^	.31^*^	.80^*^																			
Occupational deprivation	.40^*^	.38^*^	.86^*^	.61^*^																		
Occupational alienation	.48^*^	.40^*^	.80^*^	.50^*^	.66^*^																	
Occupational marginalization	.40^*^	.40^*^	.88^*^	.54^*^	.69^*^	.61^*^																
GEN-HL 0-50	.19^*^	.24^*^	.18^*^	.13^*^	.16^*^	.15^*^	.18^*^															
HC-HL 0-50	.11^*^	.14^*^	.12^*^	.10	.11^*^	.05	.13^*^	.87^*^														
DP-HL 0-50	.15^*^	.24^*^	.18^*^	.12^*^	.16^*^	.15^*^	.17^*^	.91^*^	.72^*^													
HP-HL 0-50	.24^*^	.24^*^	.18^*^	.11^*^	.16^*^	.18^*^	.17^*^	.90^*^	.65^*^	.73^*^												
TMD score	.75^*^	.73^*^	.48^*^	.35^*^	.39^*^	.46^*^	.41^*^	.19^*^	.11^*^	.15^*^	.23^*^											
A-H	.26^*^	.31^*^	.29^*^	.16^*^	.22^*^	.23^*^	.34^*^	.06	.04	.07	.04	.57^*^										
C-B	.62^*^	.56^*^	.35^*^	.28^*^	.31^*^	.31^*^	.29^*^	.22^*^	.15^*^	.17^*^	.25^*^	.86^*^	.43^*^									
D-D	.65^*^	.59^*^	.41^*^	.28^*^	.34^*^	.41^*^	.37^*^	.16^*^	.09	.11^*^	.21^*^	.88^*^	.49^*^	.75^*^								
F-I	.54^*^	.52^*^	.38^*^	.36^*^	.31^*^	.33^*^	.29^*^	.11^*^	.04	.11^*^	.13^*^	.83^*^	.39^*^	.71^*^	.68^*^							
T-A	.62^*^	.60^*^	.29^*^	.27^*^	.24^*^	.25^*^	.21^*^	.11^*^	.07	.08	.15^*^	.83^*^	.31^*^	.78^*^	.65^*^	.71^*^						
V-A	−.55^*^	−.54^*^	−.34^*^	−.18^*^	−.27^*^	−.45^*^	−.28^*^	−.17^*^	−.10	−.13^*^	−.20^*^	−.39^*^	.02	−.10	−.24^*^	−.13^*^	−.14^*^					
F	−.32^*^	−.37^*^	−.25^*^	−.07	−.20^*^	−.36^*^	−.24^*^	−.15^*^	−.10	−.15^*^	−.15^*^	−.18^*^	.02	.06	−.11^*^	.00	.07	.70^*^				
Total score	.64^*^	.65^*^	.48^*^	.35^*^	.40^*^	.41^*^	.45^*^	.14^*^	.08	.12^*^	.16^*^	.75^*^	.52^*^	.62^*^	.70^*^	.59^*^	.55^*^	−.30^*^	−.23^*^			
Depression-Anxiety	.63^*^	.65^*^	.46^*^	.34^*^	.39^*^	.38^*^	.41^*^	.10	.05	.08	.13^*^	.72^*^	.38^*^	.58^*^	.68^*^	.59^*^	.57^*^	−.32^*^	−.21^*^	.93^*^		
Displeasure and anger	.38^*^	.44^*^	.36^*^	.24^*^	.30^*^	.25^*^	.38^*^	.06	.04	.09	.03	.55^*^	.67^*^	.42^*^	.50^*^	.43^*^	.34^*^	−.10	−.14^*^	.81^*^	.65^*^	
Lassitude	.66^*^	.59^*^	.45^*^	.34^*^	.35^*^	.42^*^	.39^*^	.20^*^	.12^*^	.15^*^	.24^*^	.69^*^	.36^*^	.61^*^	.64^*^	.52^*^	.51^*^	−.35^*^	−.25^*^	.89^*^	.77^*^	.55^*^

**Notes.**

* *p* < .05, analysis using Pearson’s correlation coefficient.

## Discussion

This study revealed that among college students without any obvious medical symptom, occupational dysfunction, negative emotions, and stress responses were strongest in the group with high trait and state anxiety. Between the two groups with low trait anxiety, the group with low trait anxiety and high state anxiety showed stronger occupational dysfunction, negative emotions, and stress responses. In the present study, the prevalence of occupational dysfunction was 47% for the participants as a whole and 61% for the group with particularly high anxiety, with a significantly different distribution among the four groups according to trait and state anxiety. These results support our first research hypothesis. Occupational dysfunction can be a barrier to social participation. It can lead to a decrease in health-related quality of life of people with occupational dysfunction, as opposed to those without occupational dysfunction (Molineux, 2004). Because function and performance are interchangeable concepts in occupational therapy, issues related to occupational performance and occupational participation are recognized as the same as occupational dysfunction (Anaby et al., 2010; Bryant, Craik & McKay, 2004; Townsend & Wilcock, 2004). The COVID-19 pandemic has most recently had a major impact on all aspects of life, occupational performance, and occupational participation (Sizemore, Peganoff-O’Brien & Skubik-Peplaski, 2021). Social distancing has been emphasized from early on, such as avoiding travel, limiting physical contact with people outside the home, and maintaining a certain distance between self and others in public places. (Coroiu et al., 2020). Although detailed policies vary from country to country, social distancing restricts the scope of activities and inevitably has a negative impact on work performance and participation. Due to the COVID-19 pandemic, changes in daily life have been reported to affect health and well-being, resulting in consistent occupational changes (Fristedt et al., 2021). These changes in the social milieu and daily life lead to increased anxiety. Therefore, when considering the occupational dysfunction status of college students, it may be useful to combine the ratings of high and low state and trait anxiety.

In this study, there was a moderate correlation between occupational dysfunction, negative emotion, and stress responses. These results support our second research hypothesis. Further, the levels of anxiety and stress in the participants of the current study were highly similar to those found in participants of a previous study, which concluded that the levels of anxiety and stress of college students were high during the COVID-19 pandemic (Stamatis et al., 2021). It was also a time when the general public was aware of the need to refrain from going out, although the number of people infected with COVID-19 was not high in Japan. It is unclear to what extent the participants of this study had changes in their lives due to COVID-19 and their awareness of social trends because a survey on these topics was not conducted, but it is possible that these changes, alongside increased screen time, affected occupational dysfunction. Thus, negative emotions and stressful situations need to be carefully monitored in the progress of students with occupational dysfunction.

Encouraging occupational participation in the areas of self-care, productivity, and leisure has been reported to be effective in reducing occupational dysfunction among healthcare workers with high anxiety and stress (Teraoka & Kyougoku, 2019). Although the mental health of college students may not have changed significantly in the early stages of the pandemic, their levels of anxiety, depression, and stress were generally higher throughout that time (Stamatis et al., 2021). For college students, moderate exercise reduces anxiety and depression (Johnston et al., 2021), and mental health programs that involve talking with others reduce stress and depression (Cho & Jang, 2021). Encouraging occupational participation in self-care and leisure activities, such as moderate physical exercise, and productivity that involves interaction with others, may lead to a decrease in anxiety and stress among college students. In particular, in this study, occupational alienation showed a moderate relationship with trait anxiety and negative emotions, and occupational deprivation showed a moderate relationship with stress. In the COVID-19 pandemic survey of Japanese health care workers, 440/661 (66.6%) had lower mental health, but higher satisfaction with work and new activities (work participation) mitigated the decline in mental health (Tahara, Mashizume & Takahashi, 2021). Considering the definitions and properties of occupational alienation and deprivation (Bryant, Craik & McKay, 2004; Whiteford, 2000), it is important to facilitate an individual’s internal needs related to daily activities and to increase the opportunity for daily activities that are beyond the individual’s control. Specifically, for college students learning in a hybrid teaching format, teachers should provide assignments that encourage newly occupational participation in the area of productivity to maintain good mental health. Interventions related to occupational dysfunction prevention may be more effective if the degree of state and trait anxiety is taken into account.

People with low health literacy are more likely to suffer from various life problems such as high anxiety and increased prevalence of sleep disorders (Zhang et al., 2019). In this study, health literacy, as measured by the HLS-EU-Q47 score, was lower in the group with low trait anxiety and low state anxiety than in the group with high trait anxiety and high state anxiety. Health literacy had a low correlation coefficient with the other parameters. Thus, the results of our study differed from those of previous studies, which showed that people with low health literacy are more likely to have problems in their lives. Information about the COVID-19 pandemic provided by the media is both trusted and not trusted and has been spread rapidly to keep the public abreast of the global situation and to prevent infection (Khan et al., 2022). In the survey about COVID-19 Information Sources and Mental Health, trusting social media may worsen mental health (Patwary et al., 2021). Health literacy in Japan is lower than that in Europe because of numerous issues, such as the inefficient Japanese primary health care system and the difficulty in accessing reliable and comprehensible health information in Japan (Nakayama et al., 2015). In the HLS-EU-Q47, three subcategories are assessed for each of the four competencies related to health information: accessing, understanding, appraising, and applying (Sørensen et al., 2013; Nakayama et al., 2015). Those who have more information on the virus could have more anxiety about the process of obtaining, understanding, evaluating, and using the information. They could be more worried about the different situations, which may lead to transmission of the virus. Alternatively, they could be concerned about all the preventive measures they may have to take to avoid the virus. However, this is just a hypothesis, and more detailed research is needed. To reduce occupational dysfunction and anxiety about the COVID-19 pandemic, college students may need to discuss COVID-19 information with parents and other students, and universities and teachers may need to reduce students’ anxiety by maintaining opportunities for discussion and frequent involvement.

### Limitations

This study had some limitations. This was a cross-sectional study with a small number of participants in the group with high trait anxiety and low state anxiety; therefore, generalization of the results may be limited. In addition, since there were many items to be measured in this study, all measurements were conducted on the same participant within one week. Since the study period was approximately three weeks, some participants may have experienced some discrepancies in their mental health status. Therefore, caution should be exercised in interpreting the results of CAOD measured the first time and anxiety and stress measured the second time. It is difficult to separately investigate whether the anxiety and stress of the participants is due to the restrictions on their lives caused by COVID-19, or due to changes in the teaching system, such as hybrid classes, or a result of both these factors. In the future, it will be necessary to comprehensively investigate related personal and social factors.

## Conclusions

Among college students without any evident medical disease taking a hybrid of face-to-face and web-based distance learning classes to combat COVID-19 infection, the prevalence of occupational dysfunction was highest in the group with high trait anxiety and high state anxiety, occupational dysfunction, negative emotions; stress responses were strongest in this group. Because negative emotions and stressful situations need to be monitored in the progress of students with occupational dysfunction, our findings provide useful avenues for targeted support and more appropriate evaluation and interventions that take into account the state of occupational dysfunction.

## Supplemental Information

10.7717/peerj.13443/supp-1 Data S1 Raw data

10.7717/peerj.13443/supp-2 Supplemental Information 2 Consent form in Japanese

10.7717/peerj.13443/supp-3 Supplemental Information 3 Consent form in English

## Additional Information and Declarations

### Data Availability

The following information was supplied regarding data availability: The raw data are available in the Supplemental File and at Figshare: occupational dysfunction considering the states of anxiety during the COVID-19 pandemic in college students (dataset). Figshare. DOI: https://dx.doi.org/10.6084/m9.figshare.19068218.
