# Supplementary material for: Differences in college students’ occupational dysfunction and mental health considering trait and state anxiety during the COVID-19 pandemic
Source: PeerJ. 2022 May 19;10:e13443. doi: 10.7717/peerj.13443 (PMC9124458; doi:10.7717/peerj.13443)
Supplement: Supplemental Information 2 [file peerj-10-13443-s002.docx]

**「大学生のCOVIDパンデミック期間中の生活状況の調査」**

**に関する研究協力のお願い**

1. 研究目的

近年、ヘルスリテラシー（以下、HL）は健康に密接に関連し、健康やウェルビーングの改善に重要な要因として注目されています。HLは、情報を入手するだけではなく、情報を理解し、評価して活用できる力であり行動に映すことまでが内包された概念です。国際的なCOVIDパンデミックにおける状況で、大学の授業は遠隔化が進んでいます。本学では、遠隔授業と対面授業を組み合わせたハイブリッド授業を行っています。HLや不安状態などのメンタルヘルスや様々な作業の遂行状態が妨げられている可能性があります。大学生の生活状況や健康に関する情報を解明することが、より良いメンタルヘルスへの関わりへとつながります。

そこで、今回は、皆さんの生活状況やメンタルヘルスの状況などを調査することとなりました。

2.　研究方法

　対象者は東京工科大学に在籍する学生400名です。生活状況の調査やメンタルヘルスの調査を２回に分けて実施します。各測定にかかる時間はそれぞれ、約40分です。本調査に対する謝礼はありません。対象者には、本研究の目的を口頭と書面（本文）によってを説明します。

3. 予想される研究上の個人情報の管理について

研究によって得られた測定情報は、記載内容から個人を特定される可能性があるため、測定日中に実施責任者もしくは実施分担者が符号表を作成し、測定情報と符合表を別々のロックのかかるUSBメモリーに保存し、実施責任者もしくは実施分担者の研究室にある書棚に鍵をかけて、別々に保管し厳重に管理します。

4. 自由意思による参加と研究に参加されなくとも不利益を被らないことについて

　この研究への参加は、対象者の自由な意思で決めることができます。また、同意を拒否することによって、対象者がどんな不利益も被ることはありません。説明文書に連絡先を記入し、常に連絡をとれるようにしておきます。なお、協力していただいた場合、回答内容が成績に影響を及ぼすことはありません。

5. 同意撤回の自由について

いったん本研究に参加することに同意した後でも、自由に研究への参加を取り止めることができます。その場合でも何ら大学内の授業や生活にて不利益を被ることはありません。本研究参加に同意できない場合には、下記の連絡先に、メールをして下さい。本研究の説明文書を保護者の方々に渡し、見せて下さい。保護者の方が本研究に同意できない場合も、同様にその意向を示して下さい。論文投稿後や研究終了後の同意撤回については、対応できない場合があります。

6. 参加した対象者のプライバシー保護について

研究成果は学会発表等の学術目的のために公表されることがあります。その場合も個人情報の秘密は厳重に守られ、第三者にはわからないようにされます。なお、本研究は東京工科大学倫理委員会の承認を得て行われています。各データの保存期間は、論文発表の終了する2025年3月31日までとします。研究終了後は実施責任者がUSBメモリーを初期化しデータを破棄します。

ご不明な点がありましたら説明文書に記載されている連絡先にご連絡ください。よろしくお願いいたします。

実施責任者：東京工科大学作業療法学科　大野 勘太

連絡先：東京都大田区西蒲田5-23-22東京工科大学

TEL：03-6424-2151　　Mail：ohnoknt@stf.teu.ac.jp

実施分担者： 東京工科大学　肥後梨恵子

福島県立医科大学保健科学部理学療法学科　楠本泰士

同意書

　私は、下記の研究課題の 研究対象者 となることに同意します。

なお、同意にあたっては、説明書に基づき、下記の項目について内容を理解し、納得したうえであることを付記します。

記

研究課題名：大学生のCOVIDパンデミック期間中の生活状況の調査

研究責任者　（所属）　東京工科大学　　（氏名）　大野 勘太

＜説明を受けた項目＞

1. 研究の目的と意義
2. 研究の方法
3. この研究への参加予定期間と参加予定者数
4. この研究の対象者とさせていただいた理由
5. 研究参加により予想される利益と不利益 ・負担
6. 研究への参加の自由と同意撤回の自由について
7. 個人情報の保護・研究結果の取扱いについて
8. 試料・情報の保管及び廃棄の方法
9. ご本人の費用負担または謝礼について
10. 研究に関する情報公開の方法
11. 研究を行うことの適否の審査について
12. 研究組織

【対象者の署名欄】

　　　　　　　　　　　　　　　　同意日　　　　　　年　　　　月　　　　日

　　　　　　　　　　　　　　　　氏　名（署名）

【説明した担当者の署名欄】

説明日：　　　 年　 　　月　 　　日

　　　　　　　　　　　　　　　　　　所　属：

氏　名（署名）
