# Supplementary material for: Differences in college students’ occupational dysfunction and mental health considering trait and state anxiety during the COVID-19 pandemic
Source: PeerJ. 2022 May 19;10:e13443. doi: 10.7717/peerj.13443 (PMC9124458; doi:10.7717/peerj.13443)
Supplement: Supplemental Information 3 [file peerj-10-13443-s003.docx]

**Request for Research Cooperation on**

**「Survey of Living Conditions of College Students during the COVID Pandemic」**

1. Purpose of the study

Health literacy (HL) is closely related to health and has recently gained attention as an important factor in improving health and wellbeing. HL is a concept that encompasses not only access to information, but also the ability to understand, evaluate, and use information, and to translate this into action. In the context of the international COVID pandemic, university courses are increasingly being offered at a distance. The University offers a hybrid of distance and face-to-face classes, which may interfere with mental health, such as HL and anxiety states, as well as with the performance of various tasks. Elucidating information about college students' living conditions and health can lead to better mental health involvement.

Therefore, we have decided to survey your living conditions and mental health status.

2. Research method

The target population is 400 students enrolled at Tokyo University of Technology. The survey of living conditions and mental health will be conducted in two separate sessions. Each measurement will take approximately 40 minutes. There is no honorarium for this survey. The purpose of this study will be explained to the subjects both orally and in writing.

3. Anticipated management of personal information in the research

Since the measurement information obtained from the research may identify individuals based on the content of the description, the project leader or sub-leader should prepare a code table during the measurement day, save the measurement information and code table on a USB memory stick with a separate lock, lock the table on a bookshelf in the project leader's or sub-leader's laboratory, and store the table in the same location as the measurement information. Separate storage and strict control will be maintained.

4. voluntary participation and no disadvantages for not participating in the research

Participation in this research is voluntary and subject to the subject's free will. In addition, the subject will not suffer any disadvantages by refusing consent. We will include your contact information in the explanatory document so that you can be contacted at all times. Please note that if you cooperate, your responses will not affect your results.

5. freedom to withdraw consent

Once you have agreed to participate in this study, you are free to withdraw from participation. If you do so, you will not be disadvantaged in any way in your classes or life at the university. If you do not agree to participate in this study, please send an e-mail to the contact information below. Please give and show the parents/guardians the written explanation of this study. If parents/guardians do not agree to participate in this study, please indicate their intention as well. We may not be able to accommodate withdrawals of consent after submission of the paper or after the study has been completed.

6. protection of privacy of participating subjects

Research results may be published for academic purposes such as conference presentations. In such cases, personal information will be kept strictly confidential and will not be revealed to any third party. This research is conducted with the approval of the Tokyo University of Technology Ethics Committee. Each data will be stored until March 31, 2025, the end of the publication of the paper. After the completion of the research, the principal investigator will initialize the USB memory stick and destroy the data.

If you have any questions, please contact us at the contact information provided in the explanatory document. Thank you for your cooperation.

Project leader：Major of Occupational Therapy, Department of Rehabilitation, Tokyo University of Technology　Ohno Kanta

Contact：5-23-22 Nishikamata, Ohta-ku, Tokyo, 144-8535 JAPAN

TEL：03-6424-2151　　Mail：ohnoknt@stf.teu.ac.jp

Sub-leader：Tokyo University of Technology　 Higo Rieko

Fukushima Medical University School of Health Sciences　 Yasuaki Kusumoto

Participant consent form

I agree to be a research subject for the following research project.

In giving my consent, I also state that I understand and agree to the following items based on the explanatory document.

記

Title of Research: Investigation of the living conditions of university students during the COVID pandemic

Principal Investigator (Affiliation) Tokyo University of Technology (Name) Kanta Ohno

＜Item explained＞

1. purpose and significance of the study

2. research methods

3. expected duration and number of participants in the study

Reason for being selected as the subject of this study

5. anticipated benefits and disadvantages of participation in the study ・Burden

6. freedom to participate in research and freedom to withdraw consent

7. protection of personal information and handling of research results

8. method of storage and disposal of samples and information

9. the person's cost-sharing or honorarium

10. method of disclosing information on the research

11. review of the appropriateness of conducting research

12. research organization

【Signature of the Subject】

　　　　　　　　　　　　　　　　Date of Consent:

Year Month Day

　　　　　　　　　　　　　　　　Name (Signature)

【Signature of the person in charge who explained the situation】

　Date of explanation:

Year Month Day

　　　　　　　　　　　　　　　　　　Affiliation：

Name (Signature)
